# Bilateral Sterile Pyogranulomatous Keratitis in a Dog

**DOI:** 10.1155/2019/8516981

**Published:** 2019-08-20

**Authors:** Michael C. Rahe, Aubrey Cordray, Joseph Haynes

**Affiliations:** ^1^Department of Veterinary Diagnostic and Production Animal Medicine, Iowa State University, 1850 Christensen Dr., Ames, IA 50011, USA; ^2^Humboldt Veterinary Clinic, Humboldt, IA, USA; ^3^Department of Veterinary Pathology, Iowa State University, Ames, IA, USA

## Abstract

**Purpose:**

To describe the clinicopathologic features of bilateral sterile pyogranulomatous keratitis in a 16-year-old spayed female rat terrier dog.

**Methods:**

The dog presented one year prior due to ulceration of the right and left corneas. The ulcers healed but plaques developed on both eyes which progressed, during the course of one year, to cover both the left and the right corneas. Due to the animal's loss of sight and its painful condition, bilateral enucleation was performed with submission of the eyes for histopathology.

**Results:**

Microscopic examination revealed bilateral pyogranulomatous keratitis absent of etiological organisms.

**Conclusions:**

To the authors' knowledge, this is the first documented case of bilateral sterile pyogranulomatous keratitis in a dog.

## 1. Introduction

Pyogranulomatous inflammation is a chronic inflammatory response of both macrophages and neutrophils which can result from a variety of causes, including failure of the acute inflammatory response, unique biochemical characteristics (foreign material), and/or virulence factors in infectious agents such as* B. dermatitidis*,* Nocardia*,* Mycobacterium spp*., or* Leishmania*. Generally, this inflammation is induced by an exogenous agent which has entered the body, is recognized as foreign, and is subsequently surrounded and destroyed by reactive white blood cells. However, an etiologic agent is not always necessary, or found, to incite pyogranuloma formation. There are numerous cases of granulomatous or pyogranulomatous inflammation which are thought to be caused by immunoreactivity to an allergen or an autoantigen, including sperm granulomas, eosinophilic granuloma complex, and sterile pyogranuloma/granuloma syndrome (SPGS) [1–3]. In the eye, nodular granulomatous episcleritis (NGE) and necrotic scleritis are two conditions which are known to induce histiocytic to granulomatous inflammation in the absence of an etiologic agent [4, 5]. However, these conditions are primarily restricted to the sclera of domestic animals.

Here, we present the clinicopathologic characteristics of a 16-year-old spayed female rat terrier with a history of bilateral corneal plaques. Following therapeutic failure and progression of the plaques, bilateral enucleation and histopathology revealed the lesions to consist of sterile pyogranulomatous nodules which were restricted to the cornea. To the authors' knowledge, this is the first reported case of bilateral sterile pyogranulomatous keratitis in a dog.

## 2. Case Presentation

A 16-year-old spayed female rat terrier that had never traveled out of the state of Iowa presented to the Humboldt Veterinary Clinic in June of 2017 for squinting of the right eye (OD). There was neovascularization and congestion of the sclera OD and fluorescein stain identified two small corneal ulcers in the right (1 mm) and left (1.5 mm) eyes. Intraocular pressure was 10 mmHg OD and 9 mmHg on the left eye (OS). Topical gentocin drops were started at that time.

The dog represented four months later, in October of 2017, for continued squinting. Body temperature (99.5°F) and intraocular pressure were still normal, OD 16 mmHg and OS 19 mmHg. The corneal ulcers were no longer present, but the sclera still showed neovascularization and congestion of vessels. Gentocin eye drops were continued and oral nonsteroidal anti-inflammatories (NSAIDs), carprofen, were started. At recheck, two weeks later, the sclera of the right eye was still inflamed so a neomycin and polymyxin B sulfates with dexamethasone (.1%) eye drop were added to the treatment regimen OD.

**Figure 1:**
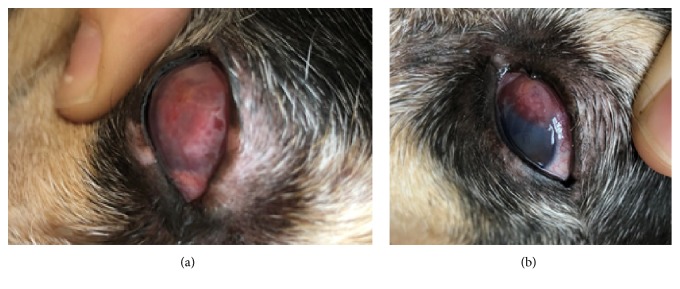
Canine-photographs of progressive plaques over the right (a) and left (b) corneas.

The dog was scheduled to be rechecked in two weeks but only presented seven weeks later, exhibiting bilateral white to red corneal plaques. The right cornea was completely covered (Figure 1(a)) while only 1/3 of the left cornea was effaced (Figure 1(b)). Differentials for the plaques included neoplasia, fungal, protozoal, or bacterial infection, or autoimmune keratitis. To rule out cultivable infectious agents, both corneas were anesthetized, the corneal plaques were gently debrided, and culture swabs of both corneas were taken for bacterial and fungal culture. Neither culture resulted in any growth. The dog was prescribed erythromycin eye drops for both eyes and continued on the oral NSAID.

The animal was rechecked five days later. Complete blood cell count (CBC) and blood chemistry (chem) were performed. The CBC showed a leukopenia, 5.06X10^9^/L (normal = 6 – 17 X10^9^/L) and mild lymphopenia, 0.89X10^9^/L (normal = 1 - 4.8 X10^9^/L). The only significant finding in the blood chemistry was a high blood urea nitrogen (BUN) 48 mg/dL (normal = 7 – 27 mg/dL); though, creatinine was normal. Prednisolone drops (1%) were prescribed for the eyes, as well as oral tramadol at a dose of 3.125 mg/kg every eight hours for pain management.

After five months of prednisolone therapy, the corneal plaques had continued to progress resulting in blindness in both eyes. As a result of this, the owners elected for bilateral enucleation. Following surgery, both eyes were submitted for histopathologic evaluation to the Surgical Pathology Service of the Department of Veterinary Pathology, Iowa State. A follow-up CBC/chem showed no abnormal findings in the CBC, while the serum chemistry panel revealed the abnormally high BUN, 47 mg/dL (normal = 7-27 mg/dL), with normal creatinine similar to previous results.

**Figure 2:**
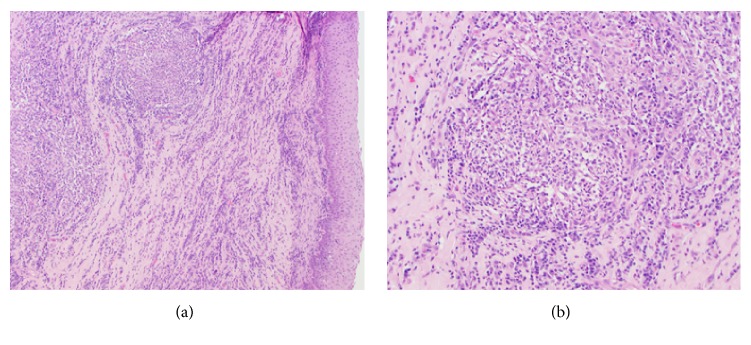
(a) Anterior cornea of the right eye (100X, H&E stain): surface epithelium is markedly hyperplastic with expansion of underlying stroma by small caliber vessels and multifocal pyogranulomatous nodules surrounded by infiltrates of lymphocytes and plasma cells. (b) Anterior cornea of the right eye (200X, H&E stain): higher magnification of previously described lesion highlighting pyogranulomatous inflammation.

Histopathologic evaluation of both eyes revealed markedly expanded corneas due to pronounced hyperplasia of the surface epithelium, along with neovascularization, fibrosis of the stroma, and a marked infiltrate of numerous distinct pyogranulomas surrounded by a diffuse infiltrate of lymphocytes and plasma cells (Figure 2(a)). The pyogranulomas were characterized by a peripheral zone of epithelioid macrophages, fewer lymphocytes and plasma cells, and a central zone of polymorphonuclear cells (Figure 2(b)). Descemet's membrane was intact but there was a layer of plasma cells and lymphocytes subjacent to the endothelium. There was a moderate perivascular infiltrate of lymphocytes and plasma cells in the iris. Also, there was a substantial infiltrate of lymphocytes and plasma cells in the bulbar conjunctiva and around the scleral vessels at the limbus. Posterior segments of the eyes were unremarkable. There was no evidence of neoplasia.

Special stains were ordered to highlight potential infectious organisms within the pyogranulomas. However, no agents were identified with Haemotoxylin and Eosin (H&E), periodic acid Schiff (PAS), Grocott's methenamine silver (GMS), Giemsa, Gram and acid-fast stains. The animal responded well to the bilateral enucleation and is doing great post-op.

## 3. Discussion

Previous reported cases of pyogranulomatous keratitis in the dog identified several protozoal organisms (ex.* Toxoplasma gondii* and* Leishmania*) as well as* Acanthamoeba* as causative agents with visualization of organisms followed by confirmatory ancillary testing [6–8]. The Beckwith-Cohen et al. protozoal keratitis cases were all identified in dogs that had received long term, topical or systemic, immunosuppressive therapy for keratoconjunctivitis sicca before masses were noted to be progressing over the corneas. In the presented case, the lesions started progressing prior to steroid treatment and continued in spite of prescribed therapy. In fact, the left eye had not received treatment of any kind prior to its development of plaques. Had the animal been infected with a protozoan,* Toxoplasma* or* Acanthamoeba* organisms likely would have been visible in one of our many sections or stains. While* Leishmania* would be more difficult to identify with histopathology, the dog has never left the state of Iowa and lives in a nonendemic area [9]. Furthermore, Giemsa stains, which have previously been shown to highlight Leishmania organisms in histopathology sections, were negative for amastigotes [10].

It is important to note that, while there was extensive pyogranulomatous inflammation in the cornea, there were also substantial populations of lymphocytes and plasma cells throughout the cornea with more mild aggregates in adjacent conjunctiva, iris, and subjacent to Descemet's membrane. This lymphoplasmacytic infiltrate was often peripheral to pyogranulomatous inflammation and could have developed in response to a number of stimuli including cytokines released from reactive macrophages, damaged keratocytes, or corneal epithelium [11, 12]. However, the lymphocytes may also be the primary reactors as can be seen in autoimmune disease and are thus responsible for pulling in additional inflammatory cells, or they may be regulatory T cells attempting to mitigate inflammation [13]. An incomplete understanding of the relationship between canine T cell coreceptor phenotype and autoimmune function/importance makes further investigation of the described lymphocytic populations difficult and likely unrewarding.

The seemingly sterile nature of these lesions would suggest that this animal developed an immune response against one of the components of the corneal stroma. This is strongly supported by the absence of pyogranulomatous inflammation in any structure of the eye besides the cornea. In general, autoimmune keratitis is rare, as the cornea is an immune privileged site [14]. Under homeostatic conditions there are numerous factors, such as anterior chamber-associated immune deviation (ACAID), that prevent naïve effector lymphocytes from responding to antigens which are normally expressed there [15, 16]. However, following injury to the eye and subsequent inflammation, this immune privilege can break down and result in an autoimmune response to proteins normally found in the anterior chamber or cornea. The result, in humans, is a sometimes markedly delayed T cell-mediated granulomatous inflammatory response of the uvea in both the injured eye (known as the exciting eye) and the uninjured contralateral eye (known as the sympathetic eye) [17]. This is known as sympathetic ophthalmia and while there are numerous publications on this phenomenon in humans, this autoimmune disease has never been described in a companion animal species [18].

Nodular granulomatous episcleritis (NGE) and necrotizing scleritis are two idiopathic inflammatory conditions of the eye characterized by numerous infiltrates of histiocytes and lymphocytes. Classically, these lesions are primarily confined to the sclera [19]. However, recent work has presented three cases of corneocentric variants of NGE [20]. It is important to note that the microscopic features of corneocentric NGE do not fit the described microscopic findings of this case. In necrotizing scleritis, lesions consist of coalescing granulomas centered on remnants of collagen with peripheral infiltrates of lymphocytes. This presentation is similar to the described case; though, we observed a large neutrophilic component to the inflammatory infiltrate and an absence of necrotic collagen within the center of granulomas. While it is possible that the presented case is a corneocentric variant of necrotizing scleritis, the disparity in microscopic characteristics warrants caution and likely requires the description of additional cases with similar features before this can be termed a true variant. As a result of this, the presented case is, to the authors' knowledge, the first reported example of bilateral pyogranulomatous keratitis in a dog without infectious stimulus or foreign substance.
